# Transcriptome analysis reveals differences in the mechanisms of fiber initiation and elongation between long- and short-fiber cotton (*Gossypium hirsutum* L.) lines

**DOI:** 10.1186/s12864-019-5986-5

**Published:** 2019-08-05

**Authors:** Yuan Qin, Huiru Sun, Pengbo Hao, Hantao Wang, Congcong Wang, Liang Ma, Hengling Wei, Shuxun Yu

**Affiliations:** State Key Laboratory of Cotton Biology, Institute of Cotton Research, Chinese Academy of Agricultural Science, Anyang, 455000 China

**Keywords:** *Gossypium hirsutum*, Fiber length, RNA-seq, Ethylene, Tubulin, Peroxidase

## Abstract

**Background:**

Improving the yield and fiber quality of upland cotton is a goal of plant breeders. However, increasing the yield and quality of cotton fibers is becoming more urgent. While the growing human population needs more cotton fiber, climate change is reducing the amount of land on which cotton can be planted, or making it difficult to ensure that water and other resources will be available in optimal quantities. The most logical means of improving yield and quality is understanding and manipulating the genes involved. Here, we used comparative transcriptomics to explore differences in gene expression between long- and short-fiber cotton lines to identify candidate genes useful for cotton improvement.

**Results:**

Light and electron microscopy revealed that the initial fiber density was significantly greater in our short-fiber group (SFG) than in our long-fiber group (LFG). Compared with the SFG fibers, the LFG fibers were longer at all developmental stages. Comparison of the LFG and SFG transcriptomes revealed a total of 3538 differentially expressed genes (DEGs). Notably, at all three developmental stages examined, two expression patterns, consistently downregulated (profile 0) and consistently upregulated (profile 7), were identified, and both were significantly enriched in the SFG and LFG. Twenty-two DEGs known to be involved in fiber initiation were detected in profile 0, while 31 DEGs involved in fiber elongation were detected in profile 7. Functional annotation suggested that these DEGs, which included *ERF1*, *TUA2*, *TUB1*, and *PER64*, affect fiber elongation by participating in the ethylene response, microtubule synthesis, and/or the peroxidase (POD) catalytic pathway. qRT-PCR was used to confirm the RNA sequencing results for select genes.

**Conclusions:**

A comparison of SFG and LFG transcription profiles revealed modest but important differences in gene expression between the groups. Notably, our results confirm those of previous studies suggesting that genes involved in ethylene, tubulin, and POD pathways play important roles in fiber development. The 22 consistently downregulated DEGs involved in fiber initiation and the 31 consistently upregulated genes involved in fiber elongation are seemingly good candidate genes for improving fiber initiation and elongation in cotton.

**Electronic supplementary material:**

The online version of this article (10.1186/s12864-019-5986-5) contains supplementary material, which is available to authorized users.

## Background

Cotton (*Gossypium* spp.) is an important fiber crop species worldwide. Among all the cultivated species, upland cotton (*Gossypium hirsutum* L.) is widely planted and accounts for more than 95% of the global cotton production. Cotton fiber is the main raw material for the textile industry, and its characteristics affect the quality of cotton textiles. Due to the growing human population and societal reasons, the demand for textiles is increasing. Moreover, owing to the serious environmental pollution associated with materials that rely on fossil resources such as petroleum, synthetic fibers do not meet the criteria for sustainable development. Therefore, the production of increased amounts of and better cotton fibers is becoming crucial.

Cotton fibers initiate from the ovule epidermis on the day of anthesis. After fiber initiation, fiber cells expand rapidly as a result of increased intracellular swelling pressure and cell wall relaxation. The fiber development process can be divided into four overlapping stages on the basis of morphological characteristics during a 50-day period: initiation (0–3 days post anthesis (DPA)), elongation (3–20 DPA), secondary cell wall thickening (16–40 DPA) and maturation (40–50 DPA) [1]. Mature fibers can be classified into two types: lint fibers and fuzz fibers. Lint fibers initiate at 0–2 DPA, and fuzz fibers initiate 2–5 days later [2].

As a heterotetraploid crop species, upland cotton has a large and complex genome [3]. Identification of the genes involved in fiber initiation and elucidation of the fiber development mechanism are hard. Cotton fibers, which constitute a type of seed hair, reportedly share similarities with leaf trichomes [4–6]. Based on the epidermal hair regulatory complex GL1-TTG1-GL2 in *Arabidopsis thaliana* [7–11], the homologous genes involved in cotton ovule epidermis were cloned and shown to restore the trait in *Arabidopsis hairless* mutants [12–14]. With the development of transgenic technology, an increasing number of genes involved in fiber development have been functionally verified. When the expression of the R2R3 MYB transcription factor *GhMYB109* was inhibited, fiber initiation was delayed, indicating that this gene is required in the initial development of fibers [15]. Overexpression of *GhMYB25* resulted in increases in the numbers of cotton fibers and leaf epidermal hairs, while gene silencing led to opposite results [16]. When the *GhMYB25-like* gene was silenced, the plants produced naked seeds but normal epidermal hairs on other parts, indicating this gene has important functions during the fiber cell differentiation stage [17]. Then, *GhMYB25-like* was found to be the mutated gene responsible for the naked seed mutant phenotype through a map-based cloning strategy [18]. In addition, silencing of *GhHD-1* reduced the number of epidermal hairs and delayed fiber initiation, and the initial fiber number increased when *GhHD-1* was overexpressed [19].

In addition to the fiber initiation associated genes mentioned above, some factors that regulate fiber elongation have been reported, such as the turgor pressure [20], sucrose [21], calcium (Ca^2+^), reactive oxygen species (ROS) and actin [22]. By activating the function of the *GhCaM7* gene, Ca^2+^ plays key roles in promoting cotton fiber elongation, while ROS has an opposite effect in this pathway [23]. *WLIM1a* and *GhCFE1A*, whose gene products are involved in actin bundles and act as dynamic linkers between the endoplasmic reticulum and actin cytoskeleton, play important roles in fiber elongation [24, 25]. Shan et al. revealed a new molecular mechanism by which *GhHOX3* promotes cotton fiber elongation [26]. Ethylene can also promote cell elongation by regulating sucrose synthase, tubulin and expansion-related proteins [27–29].

Genes are decisive factors in controlling phenotypes. Identifying genes related to fiber development has become crucial for genetic improvements of fiber. RNA sequencing (RNA-seq) techniques have been applied, and a number of fiber-related genes have been screened and identified [30, 31]. The complete whole-genome sequence of upland cotton [32, 33] has provided a reference for functional genomics research. As an effective tool to reveal gene expression differences, transcriptome sequencing represents a classic high-throughput method for identifying differentially expressed genes (DEGs) between different species or between different developmental stages [34, 35]. In the present study, we observed seed phenotypes at the fiber initiation stage, measured the length of developing fibers between short- and long-fiber cotton lines, and confirmed the difference in fiber quality. Comparative transcriptome analysis was then applied to these two cotton groups to clarify two major issues: (1) the differences in transcriptional changes associated with different developmental stages and genotypes and (2) the genetic basis for the differences in fiber quality between short- and long- fiber cotton lines. The results of this study might provide new insights into the molecular mechanisms of fiber development in upland cotton, which would help researchers explore new ways for creating additional and better fibers.

## Methods

### Plant materials and sample collection

Four *Gossypium hirsutum* lines (69–6025-12, Liao 1779, 601 long-staple cotton (LSC), J02–508) were selected in this study. The seeds of all four lines were retrieved from the national cotton germplasm mid-term bank located at the Institute of Cotton Research, Anyang, China. The fiber length (FL) of these four lines was stable across years, but there were large differences between the lines (Table 1). The FL of 69–6025-12 and Liao 1779 was always less than 26 mm; therefore, these lines composed what was defined as the short-fiber group (SFG). The FL of 601 LSC and J02–508 was always greater than 32 mm; therefore, these lines composed what was defined as the long-fiber group (LFG).

**Table 1:** Analysis of fiber traits of four cotton lines

Trait	69–6025-12^a^	Liao 1779 ^a^	601 LSC^a^	J02–508^a^	Deviation^b^
Lint percentage, %	39.30	40.90	30.50	31.50	9.10**
Fiber length, mm	25.77	25.63	33.00	32.50	−7.05***
Fiber length uniformity, %	81.40	80.10	85.57	86.80	−5.43***
Fiber strength, cN/tex	24.53	24.17	34.93	40.53	−13.38***
Micronaire reading	5.23	4.50	2.77	4.03	1.47**
Fiber elongation, %	6.53	6.50	7.00	7.00	−0.48***

^a^Three biological replicates were measured per line. The data presented in the table are the average values. ^b^The deviation (D) was calculated as D = Mean (69–6025-12 + Liao 1779) - Mean (601 LSC + J02–508); ‘Mean’ means taking the average. **indicates significance at a probability of 0.01; ***indicates significance at a probability of 0.001. Two-tailed Student’s *t*-tests were used

Flowers were tagged on the day of anthesis, and sample collection was performed at − 3, 0, 3, 5, and 10 DPA. The − 3 DPA samples were collected based on bud length. The ovules and fibers were dissected immediately after the bolls were harvested, frozen in liquid nitrogen, and stored at − 80 °C. Three biological replicates were performed for every sample.

### Phenotypic observation of developing fibers

At the lint fiber initiation stage, 0 and 1 DPA ovules were observed with scanning electron microscopy. Three ovules were randomly selected from each material, and the number of bulged cells within a unit area of 0.02 mm^2^ (0.1 mm × 0.2 mm) in the middle of the surface was counted. At the fuzz fiber initiation stage, 3 DPA ovules were collected for paraffin sections. The detailed processes of scanning electron microscopy and paraffin sectioning were performed as reported by Liu et al. [36, 37]. At the fiber elongation stage, 10 DPA ovules were boiled and combined with running water, after which the length of attached fibers was measured [23]. Mature fibers were harvested by hand, and five fiber quality traits were evaluated [38].

### RNA extraction, library construction and transcriptome sequencing

The plant tissues were finely ground under liquid nitrogen. An RNAprep Pure polysaccharide polyphenol plant total RNA extraction kit (DP441, TIANGEN, Beijing) was used, and the operation steps were in strict accordance with the instructions. One microliter of the extracted RNA was subjected to 1% agarose gel electrophoresis to check the RNA integrity. In addition, the RNA concentration and contamination were measured by a Nanodrop 2000 spectrophotometer (Thermo, USA).

According to the two cotton groups and four representative stages/tissues, we mixed equal quantities of RNA within groups and at the same stage. The sample mixing scheme is shown in Table 2. This operation aimed to highlight the differences between two groups and between the different fiber developmental stages. In total, 16 cDNA libraries comprising 2 cotton groups at 4 developmental stages/tissues (Table 2) and 2 biological replicates were sequenced on an Illumina HiSeq 2500 sequencing platform, and 125 bp/150 bp paired-end reads were generated.

**Table 2:**
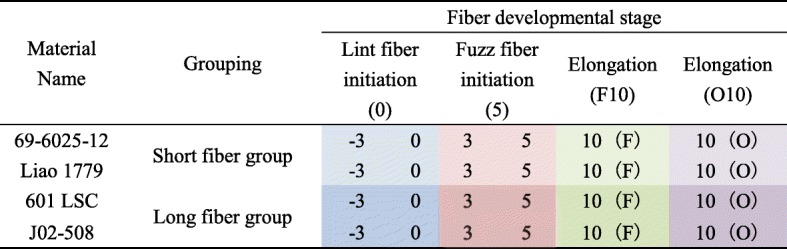
Sample mixing scheme for transcriptome sequencing

Grouping: Short fiber group, i.e., 69–6025-12 + Liao 1779; long fiber group, i.e., 601 LSC + J02–508. Fiber developmental stage: The numbers in the table indicate the days post-anthesis (DPA). In parentheses, 0 means lint fiber initiation stage, i.e., − 3 DPA to 0 DPA; 5 means fuzz fiber initiation stage, i.e., 3 DPA to 5 DPA. F10 and O10, 10 means 10 DPA, F means fiber, O means ovule. Numbers in the same color indicate that their corresponding materials and tissues were mixed equally according to RNA mass

### Bioinformatics analysis

We performed all bioinformatics analyses in accordance with standard procedures. The raw data in FASTQ format were first processed to obtain clean reads, and then the Q20, Q30 and GC content of the clean reads were calculated. Second, *Gossypium hirsutum* TM-1 genome [33] and gene model annotation files (CottonGen database, http://www.cottongen.org) were used for compiling a reference genome for clean read alignments. Third, HTSeq software [39] was used to count the number of reads mapped to each gene, and the fragments per kilobase of transcript per million mapped reads (FPKM) was calculated for estimating gene expression levels. The genes with an adjusted *p* value < 0.05 and a |log_2_ (fold change)| > 1 were then assigned as differentially expressed. In addition, personalized Gene Ontology (GO) enrichment analysis, pathway enrichment analysis, gene expression pattern analysis and principal component analysis (PCA) were performed using OmicShare tools, a free online platform for data analysis (www.omicshare.com/tools). The detailed calculation methods and parameter settings involved in the above analysis are described in Additional file 1.

### Validation of RNA-seq by qRT-PCR

To validate the RNA-seq data, 12 DEGs from the pathway enrichment analysis were selected for qRT-PCR analysis. The specific primers of these genes directly referenced the data from qPrimerDB (https://biodb.swu.edu.cn/qprimerdb) [40]. Reverse transcription and qRT-PCR were performed as previously described [41]. To properly evaluate PCR amplification efficiency, we performed 3 parallel replicates using five templates of 2-fold serial gradient dilutions first [42]. Then three technical replicates and three biological replicates were performed on all reactions. The ΔΔCt algorithm was used for calculating relative gene expression. The endogenous reference gene used was *GhActin7* (AT5G09810). All primer pairs used in the analysis and their amplification efficiencies are shown in Additional file 2.

## Results

### Differences in fiber phenotypes between long- and short-fiber cotton lines

**Fig. 1:**
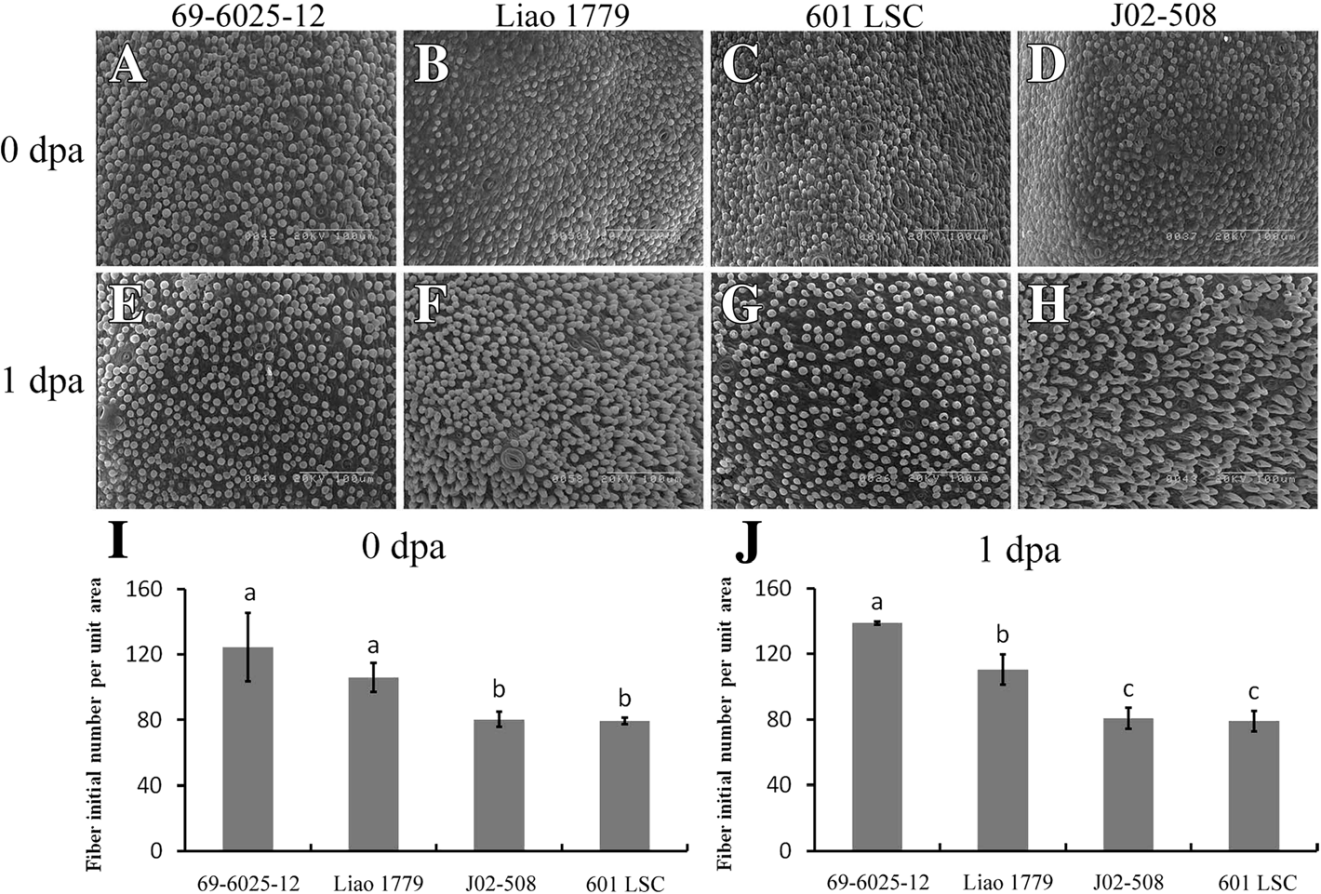
Scanning electron microscopy images of 0 DPA and 1 DPA ovules illustrating fiber initiation on the ovule surface. This image shows four upland cotton lines, i.e., 69–6025-12 (**a**, **e**), Liao 1779 (**b**, **f**), 601 LSC (**c**, **g**) and J02–508 (**d**, **f**). Scale bars: 100 μm (**a**-**h**). **i** Statistical results of the initial fiber number per unit area at 0 DPA. **j** Statistical results of the initial fiber number per unit area at 1 DPA. The height of the column is the average of three counts, and the error bars indicate ± S.D. The columns in the graph are arranged from large to small. The letters above the columns indicate the significance level of the initial fiber numbers of the four cotton materials. The same letters indicate no significant difference, and different letters indicate the difference was significant

The fiber phenotypes of the SFG (69–6025-12, Liao 1779) and LFG (601 LSC, J02–508) were different at the initiation, elongation and maturation stages. The ovule epidermal cells began to bulge at 0 DPA (Fig. 1a-d). One day later, the number of bulged cells and the cell diameter peaked (Fig. 1e-h). The average cell number of the four materials, from high to low, was in the order of 69–6025-12, Liao 1779, J02–508 and 601 LSC; the average numbers were 124.3, 106, 80.3 and 79.3, respectively, at 0 DPA (Fig. 1i), and the average numbers were 139, 110.3, 80.6 and 79, respectively, at 1 DPA (Fig. 1j). Multiple hypothesis test results showed that the differences between the SFG and LFG were significant both at 0 DPA and 1 DPA; i.e., the initial fiber numbers in the SFG were greater than those in the LFG.

**Fig. 2:**
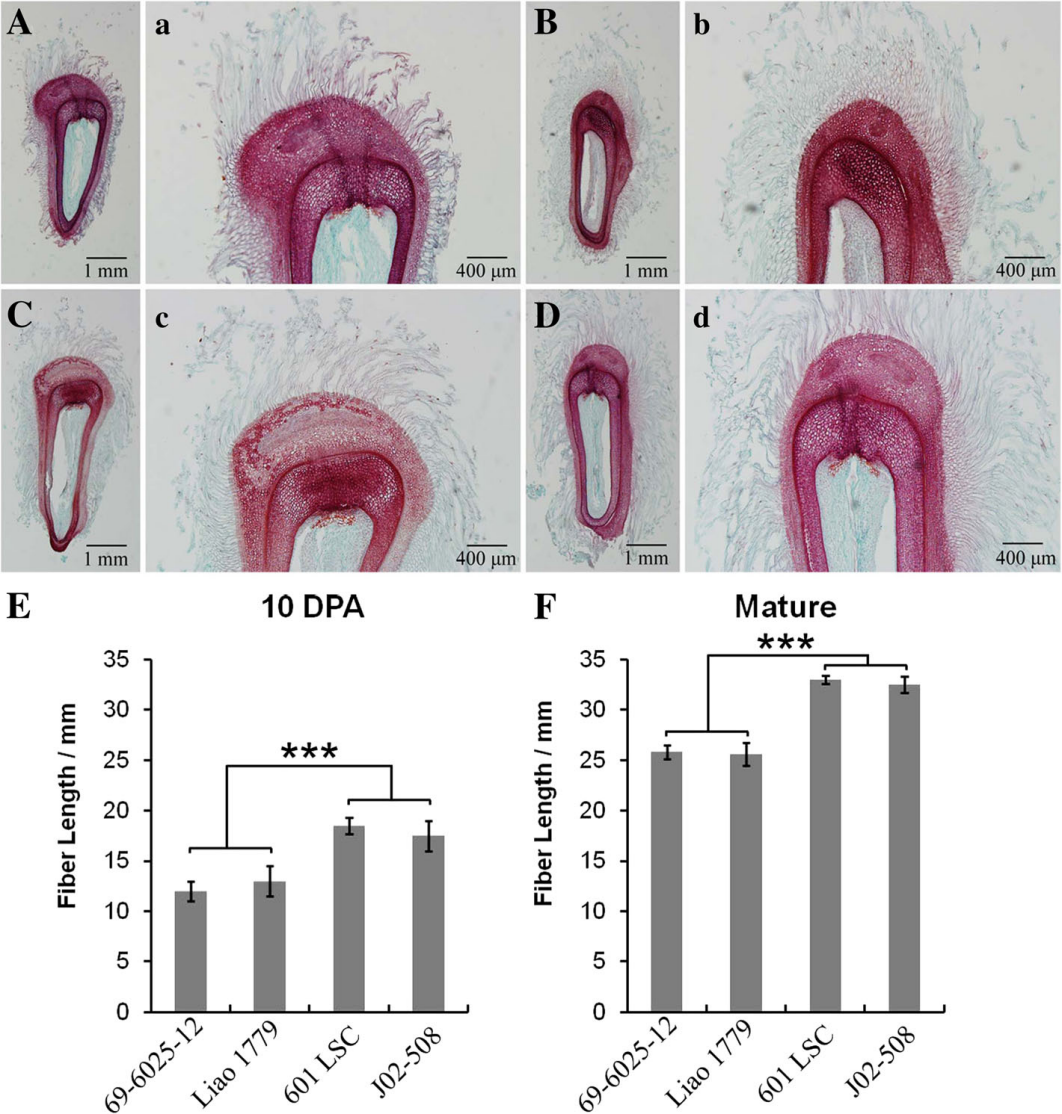
Fiber phenotype observations of four upland cotton lines. Median longitudinal sections of 3 DPA ovules illustrating the extent of fiber coverage on the surface of a seed: (**A**, a) 69–6025-12; (**B**, b) Liao 1779; (**C**, c) 601 LSC; (**D**, d) J02–508. Scale bars: 1 mm (**A**-**D**); 400 μm (a-d). **E** Difference in FL at 10 DPA between the two cotton groups. Three biological replicates were measured per material. The error bars represent the means ± S.D.s, *p* = 2.38E-05. **F** Difference in mature FL between the two groups of cotton lines. Three fiber samples were measured. The error bars represent the means ± S.D.s, *p* = 1.02E-08. (***, *p* < 0.001; two-tailed Student’s t-test)

The fiber cells elongated slightly at 3 DPA. The fiber cells of 69–6025-12 and Liao 1779 were shorter through the longitudinal section (Fig. 2a, b), while those of 601 LSC and J02–508 were longer (Fig. 2C, D). The fiber densities differed when the ovule chalazal end was further enlarged. Fibers of 69–6025-12 and Liao 1779 were arranged closely (Fig. 2A, B); there was almost no gap between fiber filaments, and the density was very high. In 601 LSC and J02–508, scattered filaments can be seen with relatively clear voids, and the fiber density was relatively low (Fig. 2C, D). This difference was consistent with the bulged cell numbers observed at 0 and 1 DPA (Fig. 1).

At 10 DPA, the FL of 69–6025-12 was 12.0 mm, and that of Liao 1779 was 13.0 mm; in contrast, the FL of 601 LSC and J02–508 was 18.5 mm and 17.5 mm, respectively (Fig. 2E). Two sets of data were averaged and then subjected to a two-tailed t-test. The results showed that there was a significant difference in FL between the two groups (Fig. 2E).

After fiber maturation, the FL of the four materials was measured; the values were 25.77 mm, 25.63 mm, 33.00 mm, and 32.50 mm (Fig. 2F, Table 1). The difference between the SFG and the LFG was extremely significant according to the results of the t-test. In addition to FL, five other fiber traits (lint percent, uniformity, fiber strength, micronaire and elongation rate) were also analyzed. In general, the differences in all six traits between the two groups were extremely significant (Table 1). Among these traits, a positive correlation was found between lint percent and micronaire value, indicating that a high micronaire value (coarse fiber) will lead to an increase in lint percent, which is consistent with the published results [43]. However, lint percent was negatively related to FL, uniformity, strength and elongation (Table 1).

Phenotypic observations at each developmental stage suggest that significant differences in the final fiber quality of the four materials were formed by the gradual accumulation of differences during fiber initiation and elongation. Therefore, these four materials might constitute a good model for revealing the molecular mechanism governing differences in fiber initiation and elongation.

### Transcriptome sequencing and quality assessment

A total of 16 cDNA libraries were constructed and sequenced in this study. The ratio of clean reads to the total number of raw reads was 94.94 to 96.44% (Additional file 3), indicating that the proportion of low-quality data in the raw reads was very low. Moreover, the proportions of sequences that could be mapped to the reference genome exceeded 85.54% in all samples, indicating no contamination and that the reference genome was appropriate. The percentage of multiple mapped reads was less than 10%, which met the criteria. In addition, Q30 ≥ 91.83% and GC contents between 43.01~45.03% were identified in all samples (Additional file 3). The data above showed that the transcriptome sequencing was of high quality.

Based on the number of genes expressed at different levels, the threshold by which genes with an FPKM > 1 (indicating that those genes were expressed) reached more than 50% in all samples (Additional file 4). Similarities greater than 97% between two biological replicates (Additional file 5) were then analyzed by the Pearson correlation coefficient method, indicating that our experiment had good repeatability. Moreover, the similarity between the SFG and the LFG at the same developmental stage reached 94% (Additional file 5) and was greater than the threshold of 92%, which indicated that our experimental operations were qualified. The gene expression became increasingly dissimilar to that of the lint fiber initiation stage (SFG-0 and LFG-0) in the subsequent two stages in both the SFG (SFG-5 and SFG-F10) and the LFG (LFG-5 and LFG-10) (Additional file 5). These results indicated that there is a dramatic change in gene expression patterns as the developmental stage changes. However, compared with the stage 0 (SFG-0 and LFG-0) and stage 5 samples (SFG-5 and LFG-5), the 10 DPA ovules (SFG-O10 and LFG-O10) were more strongly correlated than were the 10 DPA fibers (SFG-F10 and LFG-F10). This phenomenon may be related to the fibers and ovules in the previous two stages not separating. Further confirming the relationship between the two groups at different developmental stages, the PCA showed that the same developmental stage of the four materials clustered together, indicating the difference between developmental stages was greater than that between materials (Additional file 6).

### Analysis of DEGs

**Fig. 3:**
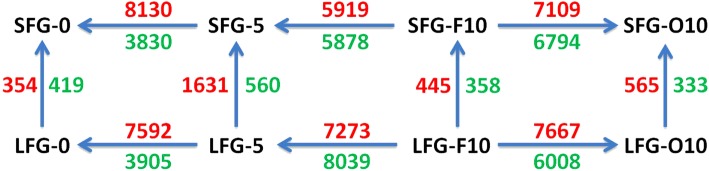
Multiple comparisons between the SFG and LFG at various stages of fiber development. The numbers around the arrows denote the numbers of genes differentially expressed for the specified comparison. Red, upregulation; green, downregulation

According to the DEG identification criteria, 773, 2191, 803 and 898 DEGs were identified at four developmental stages between the LFG and SFG (Fig. 3 and Additional file 7). The greatest number of DEGs occurred at the fuzz fiber initiation stage (5), which may be related to this stage including both fuzz fiber initiation and lint fiber elongation. Additional DEGs were identified between different developmental stages (tissues) within the material (Fig. 3). These findings showed that samples at different stages indeed dramatically changed, which was consistent with our PCA results (Additional file 6).

Cluster analysis of all the DEGs was performed using FPKM values (Additional file 8). The expression patterns were similar between the SFG and LFG but different between stages. However, the patterns at stage 5 and stage O10 were relatively similar and consistent with the Pearson correlation coefficient analysis results (Additional file 5). Moreover, gene expression was continuously upregulated or downregulated concurrently with the developmental process, which may help to reveal the mechanism governing phenotypic differences during fiber initiation and elongation.

### Gene expression pattern analysis

**Fig. 4:**
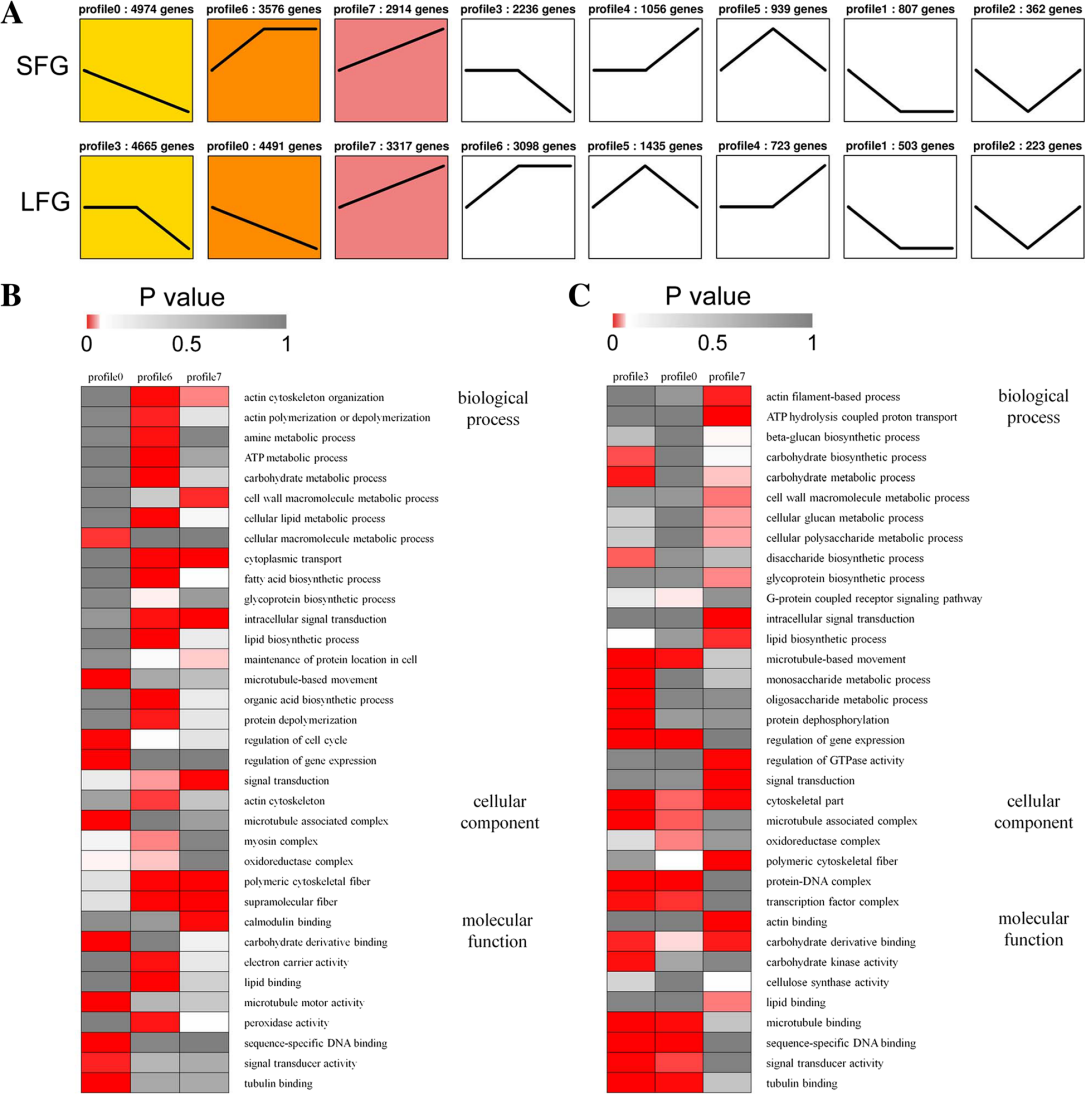
Patterns of gene expression and GO enrichment of DEGs across three developmental stages in the SFG and LFG. **a** Patterns of gene expression across three developmental stages in the SFG and LFG inferred by Short Time-series Expression Miner (STEM) analysis. Each square represents a trend of gene expression. The text on the square indicates the profile ID number and the number of genes within that profile. The black line represents the expression tendency of all the genes. A colored square indicates that the pattern was significantly enriched (*p* < 0.05). The profiles were ordered based on the number of genes enriched therein. **b** GO enrichment analysis of three significant patterns in the SFG. **c** GO enrichment analysis of three significant patterns in the LFG. The significance of the most represented GO terms in each profile is indicated by the *p* value. The red areas represent significant enrichment (*p* < 0.05), whereas the dark gray areas represent nonsignificance (*p* > 0.05)

The expression patterns of the DEGs among three developmental stages (0, 5, F10) (Additional file 7) in the SFG and LFG were analyzed (Fig. 4a). In the SFG, most DEGs were enriched in profile 0 (4974 genes, accounting for 26.00% of 19128 DEGs), followed by profile 6 (3576 genes, 18.70%) and profile 7 (2914 genes, 15.23%). In the LFG, most DEGs were enriched in profile 3 (4665 genes, 22.43% of 20795 DEGs), followed by profile 0 (4491 genes, 21.60%) and profile 7 (3317 genes, 15.95%).

The DEGs in profile 0 and profile 7 were significantly enriched in both the SFG and LFG (Fig. 4a). The expression of DEGs in profile 0 was downregulated continuously, while that in profile 7 was upregulated continuously. At the fiber initiation stage, related genes are highly expressed, but their expression levels are downregulated as development progresses [16, 17, 19]. This finding is consistent with the trend exhibited by profile 0, indicating that DEGs enriched in profile 0 are involved in the regulation of fiber initiation. However, the genes related to fiber elongation were highly expressed at 10 DPA, but their expression was lower during the early developmental stages [23, 24, 26]. These findings are consistent with the pattern of profile 7, indicating that DEGs enriched in profile 7 might be involved in fiber elongation.

Four hundred eighty-three more DEGs were enriched in profile 0 in the SFG than in the LFG (4974 vs. 4491) (Fig. 4a); i.e., more genes were involved in fiber initiation in the SFG than in the LFG. This finding was consistent with the previous result by which the SFG (69–6025-12, Liao 1779) had more bulged epidermal cells at 0 DPA and 1 DPA than did the LFG (Fig. 1). Compared with profile 0, profile 7 had 402 fewer DEGs enriched in the SFG than in the LFG (2914 vs. 3317) (Fig. 4a); i.e., more genes were involved in fiber elongation in the LFG than in the SFG. This finding was also consistent with the result by which the FL of the LFG was greater than that of the SFG at 10 DPA (Fig. 2E). Most gene expression patterns during fiber development were similar between the SFG and LFG, but the main difference lied in the number of genes and the function of key genes therein.

### Enrichment analysis

GO enrichment analysis was performed on the genes within each profile (Additional file 9). In the SFG, genes within profile 0 were enriched mainly in the cellular macromolecular metabolic process (GO:0044260, *p* = 1.05E-02), microtubule-based movement (GO:0007018, *p* = 1.52E-09), the microtubule-associated complex (GO:0005875, *p* = 9.45E-10), microtubule motor activity (GO:0003777, *p* = 1.37E-09), sequence-specific DNA binding (GO:0003700, *p* = 2.58E-15), and signal transducer activity (GO:0004871, *p* = 6.85E-03). The genes within profile 7 were enriched mainly in the cell wall macromolecule metabolic process (GO:0044036, *p* = 8.28E-03), intracellular signal transduction (GO:0035556, *p* = 5.85E-05), polymeric cytoskeletal fibers (GO:0099513, *p* = 7.30E-07) and calmodulin binding (GO:0005516, *p* = 1.66E-03) (Fig. 4b). In the LFG, the significantly enriched terms in profiles 0 and 7 are roughly consistent with the corresponding profiles in the SFG, with the exception of some specifically enriched terms, such as the oxidoreductase complex (GO:1990204, *p* = 2.55E-02) in profile 0 and ATP hydrolysis-coupled proton transport (GO:0015991, *p* = 3.23E-05), regulation of GTPase activity (GO:0043087, *p* = 5.01E-04), and carbohydrate derivative binding (GO:0097367, *p* = 5.15E-03) in profile 7 (Fig. 4c).

**Fig. 5:**
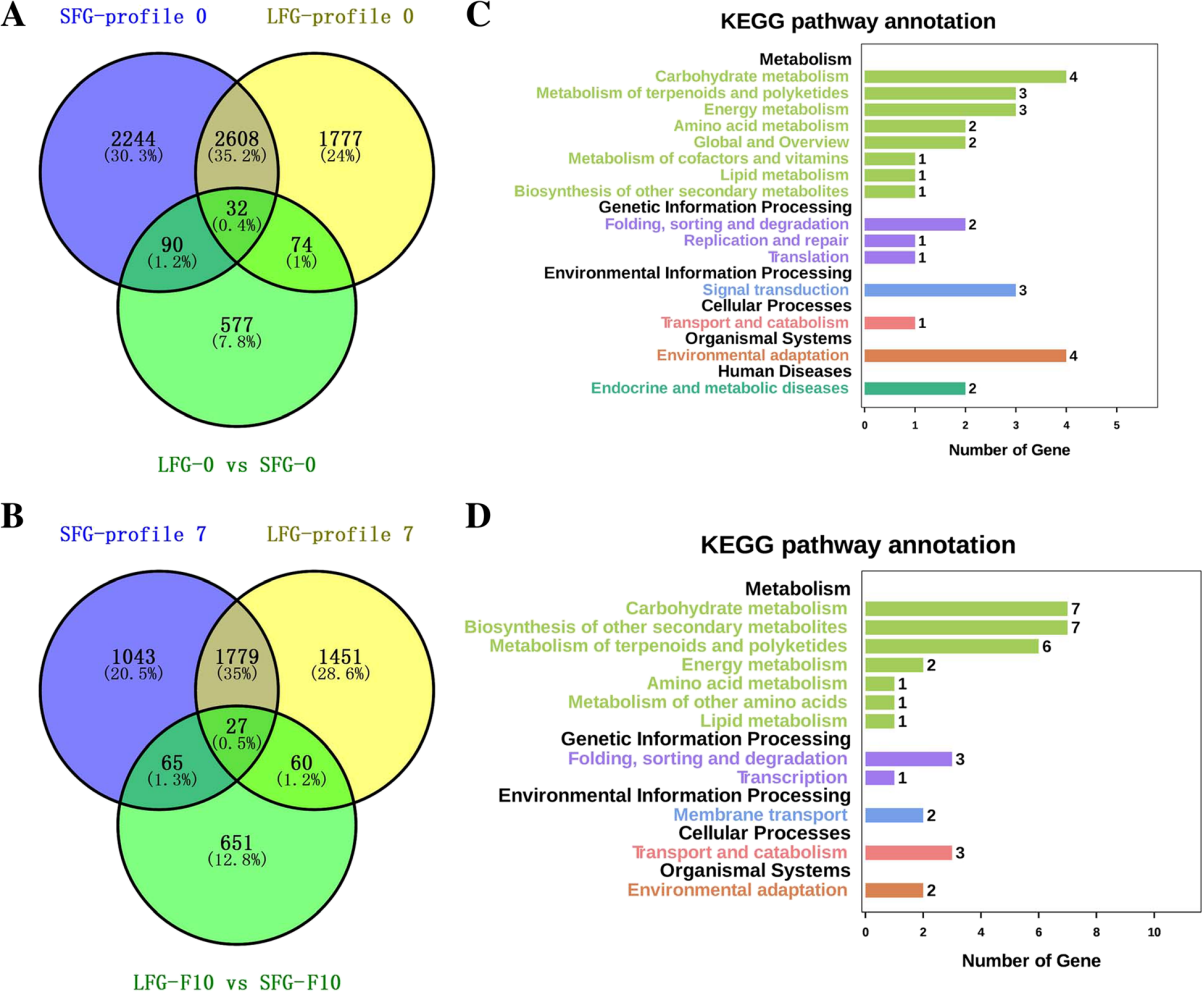
Summary of DEGs identified in this study. **a** Venn diagram showing the number of DEGs shared between profile 0 in the SFG and LFG and LFG-0 vs SFG-0. **b** Venn diagram showing the number of DEGs shared between profile 7 in the SFG and LFG and LFG-F10 vs SFG-F10. **c** KEGG pathway annotation of 196 DEGs (90 + 32 + 74) from A. **d** KEGG pathway annotation of 152 DEGs (65 + 27 + 60) from B

Previous results have shown that genes in profile 0 are associated with fiber initiation. Therefore, DEGs between SFG-0 and LFG-0 (Fig. 3, 354 + 419 DEGs) together with genes within SFG-profile 0 and LFG-profile 0 were analyzed (Fig. 5a). A total of 196 genes (90 + 32 + 74) were downregulated in the SFG or LFG and differentially expressed during the fiber initiation stage (Fig. 5a). Similarly, we analyzed the DEGs between SFG-F10 and LFG-F10 (Fig. 3, 445 + 358 DEGs) together with the genes within SFG-profile 7 and LFG-profile 7 (Fig. 5b). There were 152 genes (65 + 27 + 60) upregulated in the SFG or LFG and differentially expressed during the fiber elongation stage (Fig. 5b). To confirm which metabolic pathways the DEGs were involved in, Kyoto Encyclopedia of Genes and Genomes (KEGG) enrichment analysis was then performed on 196 and 152 DEGs (Additional file 10 and Fig. 5c, d). Twenty-two DEGs participated in different metabolic pathways during the fiber initiation (Fig. 5c), while 31 DEGs were active during the fiber elongation (Fig. 5d). Functional annotations of these 53 DEGs were obtained by homologous comparison to entries within The *Arabidopsis* Information Resource database (TAIR, http://www.arabidopsis.org/). Six different KEGG classifications were obtained, each containing at least one DEG (Table 3).

**Table 3:** DEGs with homologs in *Arabidopsis* according to KEGG pathway analysis

*G. hirsutum* gene ID	*Arabidopsis* ID	ArabDesc	Gene name	Log_2_(LFG/SFG)		
				0	5	F10
profile 0						
*Metabolism*						
Gh_A13G0351	AT3G19270	cytochrome P450, family 707, subfamily A, polypeptide 4	*AT3G19270*	−2.30	0.22	-^a^
Gh_D13G0395	AT3G19270	cytochrome P450, family 707, subfamily A, polypeptide 4	*AT3G19270*	−2.14	−0.34	− 0.17
Gh_D08G1683	AT1G32200	phospholipid/glycerol acyltransferase family protein	*ATS1*	−1.13	−0.31	− 1.08
Gh_A03G1487	AT2G43090	aconitase/3-isopropylmalate dehydratase protein	*SSU1*	−1.11	−2.40	−0.32
Gh_A01G1270	AT2G01290	ribose-5-phosphate isomerase 2	*RPI2*	1.03	1.37	−0.06
**Gh_D08G0936**^**b**^	**AT2G18700**	**trehalose phosphatase/synthase 11**	***TPS11***	**1.03**	**2.58**	**−1.43**
Gh_D01G2232	AT5G01530	light harvesting complex photosystem II	*LHCB4.1*	1.22	1.72	1.14
Gh_D01G0156	AT2G20340	pyridoxal phosphate (PLP)-dependent transferases superfamily protein	*AT2G20340*	1.25	0.92	0.58
Gh_D01G0629	AT5G36110	cytochrome P450, family 716, subfamily A, polypeptide 1	*AT5G36110*	1.32	1.50	–
Gh_D05G1605	AT5G54770	thiazole biosynthetic enzyme, chloroplast (ARA6) (THI1) (THI4)	*THI4*	1.36	0.89	−0.21
Gh_D05G2361	AT4G10340	light-harvesting complex of photosystem II 5	*LHCB5*	1.49	0.69	3.58
*Genetic Information Processing*						
Gh_A07G1168	AT4G02070	MUTS homolog 6	*MSH6*	−1.06	−0.30	−0.57
Gh_D10G0234	AT2G33770	phosphate 2	*UBC24*	1.14	1.48	0.45
Gh_A13G0294	AT2G21660	cold, circadian rhythm, and RNA binding 2	*CCR2*	1.18	0.72	−1.08
Gh_D06G1571	AT2G04030	chaperone protein htpG family protein	*HSP88.1*	2.23	2.63	2.12
*Environmental Information Processing*						
Gh_D03G0317	AT5G10570	basic helix-loop-helix (bHLH) DNA-binding superfamily protein	*AT5G10570*	−1.31	−0.63	−0.70
Gh_A06G1440	AT3G08510	phospholipase C 2	*AT3G08510*	1.02	1.79	1.58
**Gh_D11G0426**	**AT4G17500**	**ethylene responsive element binding factor 1**	***ERF-1***	**1.75**	**3.54**	**0.00**
*Cellular Processes*						
**Gh_A07G1077**	**AT5G12250**	**beta-6 tubulin**	***TUB6***	**1.01**	**1.31**	**0.62**
*Organismal Systems*						
Gh_Sca013634G01	AT5G24470	pseudoresponse regulator 5	*PRR5*	1.24	1.16	−0.84
Gh_D05G3492	AT5G54470	B-box type zinc finger family protein	*BBX29*	1.47	1.55	1.00
***Diseases***						
Gh_D08G2277	AT5G28770	bZIP transcription factor family protein	*AT5G28770*	1.08	0.83	0.71
profile 7						
*Metabolism*						
Gh_A12G0583	AT3G11480	S-adenosyl-L-methionine-dependent methyltransferases superfamily protein	*BSMT1*	2.32	1.46	−3.38
**Gh_A02G1663**	**AT5G42180**	**POD superfamily protein**	***PER64***	**−1.22**	**−1.01**	**−3.13**
Gh_A08G2395	AT2G45550	cytochrome P450, family 76, subfamily C, polypeptide 4	*CYP76C4*	−3.64	−5.57	−2.02
Gh_A11G0380	AT1G74920	aldehyde dehydrogenase 10A8	*ALDH10A8*	−0.44	−0.26	−1.98
**Gh_A05G3328**	**AT4G33440**	**PL-like superfamily protein**	***AT4G33440***	**−0.29**	**−0.91**	**−1.59**
Gh_D07G0692	AT5G15490	UDP-glucose 6-dehydrogenase family protein	*UGD3*	0.51	−1.34	−1.54
Gh_D07G2503	AT3G01910	sulfite oxidase	*SOX*	−0.27	0.24	−1.06
**Gh_A11G0965**	**AT1G02310**	**glycosyl hydrolase superfamily protein**	***AT1G02310***	**−1.29**	**0.51**	**−1.04**
Gh_A06G1309	AT4G22010	SKU5 similar 4	*SKS4*	−0.08	0.53	1.02
**Gh_D11G0237**	**AT4G23820**	**PL-like superfamily protein**	***AT4G23820***	**0.29**	**−0.07**	**1.13**
Gh_A13G1123	AT3G26330	cytochrome P450, family 71, subfamily B, polypeptide 37	*CYP71B37*	−0.34	−0.17	1.20
**Gh_A05G3108**	**AT5G40390**	**raffinose synthase family protein**	***RS5***	**0.46**	**0.21**	**1.35**
**Gh_D12G2437**	**AT3G48950**	**PL-like superfamily protein**	***AT3G48950***	**1.22**	**0.63**	**1.36**
**Gh_D11G1910**	**AT2G23620**	**methyl esterase 1**	***MES1***	**−0.05**	**0.25**	**1.52**
Gh_A12G0098	AT4G35420	dihydroflavonol 4-reductase-like 1	*DRL1*	−0.81	1.07	1.68
Gh_A05G0525	AT5G24910	cytochrome P450, family 714, subfamily A, polypeptide 1	*CYP714A1*	−0.40	1.87	1.71
Gh_D10G1008	AT4G39490	cytochrome P450, family 96, subfamily A, polypeptide 10	*CYP96A10*	0.06	−0.43	1.74
Gh_D05G0646	AT5G24910	cytochrome P450, family 714, subfamily A, polypeptide 1	*CYP714A1*	−0.70	0.54	1.80
Gh_A07G1593	AT1G65820	microsomal glutathione s-transferase, putative	*AT1G65820*	−0.81	− 0.13	1.86
Gh_D05G1149	AT1G80660	H(+)-ATPase 9	*AT1G80660*	−0.54	1.08	2.86
Gh_A09G1848	AT5G07990	cytochrome P450 superfamily protein	*CYP75B1*	–	–	3.90
Gh_A09G2349	AT1G05160	cytochrome P450, family 88, subfamily A, polypeptide 3	*CYP88A3*	1.58	3.70	4.12
***Genetic Information Processing***						
Gh_D09G1479	AT5G02500	heat shock cognate protein 70–1	*HSP70–1*	0.28	0.11	1.51
Gh_A05G2014	AT3G24590	plastidic type I signal peptidase 1	*AT3G24590*	−0.09	1.31	1.79
Gh_D05G2258	AT3G24590	plastidic type I signal peptidase 1	*AT3G24590*	1.32	0.42	2.62
*Environmental Information Processing*						
Gh_A12G1090	AT4G01820	P-glycoprotein 3	*AT4G01820*	−1.42	−0.23	1.51
Gh_D12G1213	AT4G01820	P-glycoprotein 3	*AT4G01820*	−0.81	0.51	2.26
*Cellular Processes*						
**Gh_D01G0939**	**AT1G75780**	**tubulin beta-1 chain**	***TUB1***	**−0.01**	**−0.04**	**1.20**
**Gh_D02G2420**	**AT1G50010**	**tubulin alpha-2 chain**	***TUA2***	**0.72**	**0.75**	**1.39**
*Organismal Systems*						
Gh_D07G2384	AT4G29810	MAP kinase kinase 2	*MKK2*	1.00	−2.61	−3.56
Gh_A09G2314	AT1G06040	B-box zinc finger family protein	*BBX24*	1.00	−0.49	1.02

^a^-, not detected at that developmental stage. ^b^Bold lines mean that the gene was selected for expression analysis

**Fig. 6:**
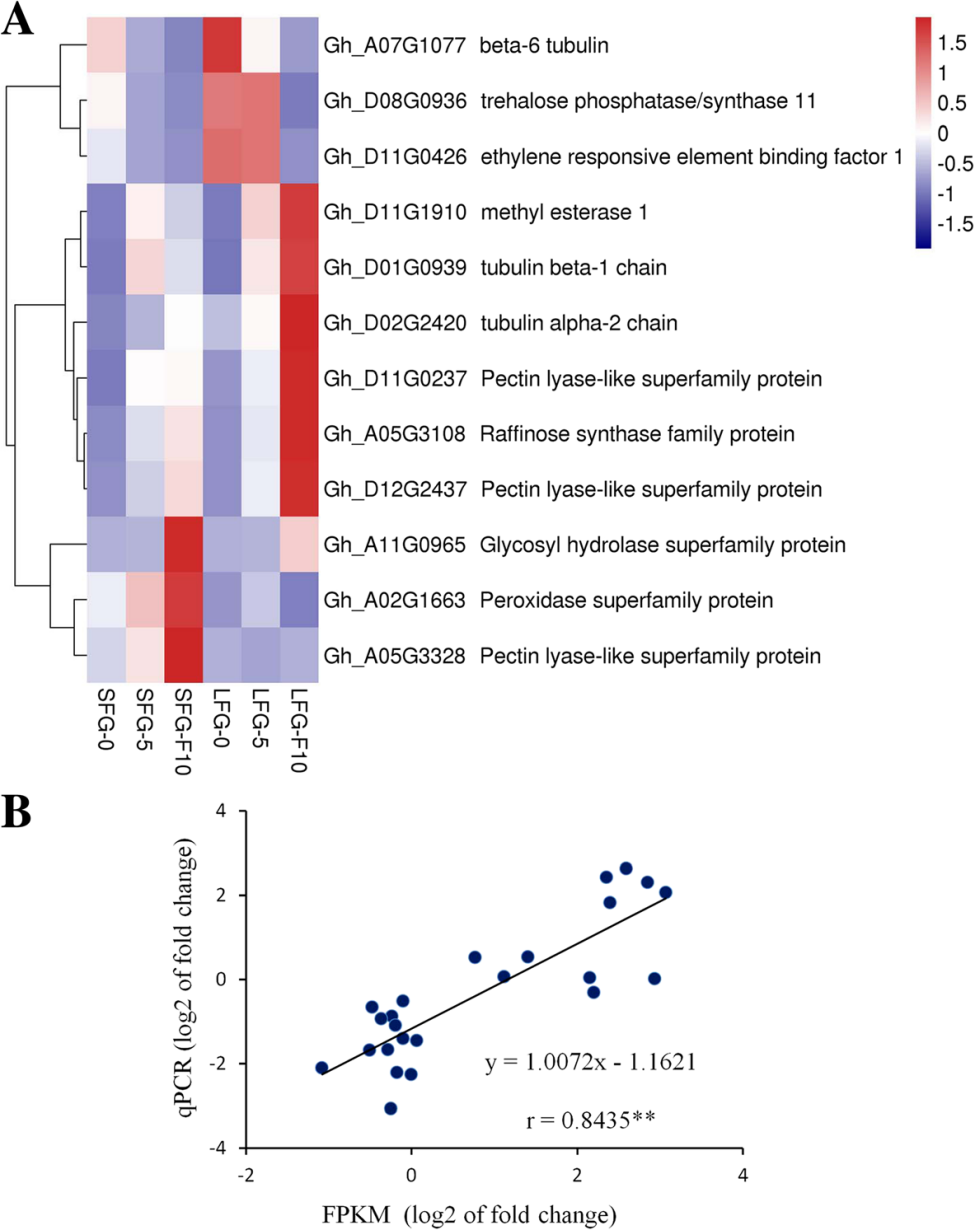
Expression analysis and verification of DEGs. **a** A heat map of 12 DEGs from profile 0 and profile 7. **b** Correlations between qPCR and RNA-seq results for the 12 selected genes. Each point represents a fold change in expression level at F10 compared with 0. The fold change values were log2 transformed

Twelve genes, which might be related to fiber development, were selected for expression analysis on the basis of their functional annotation. After they were clustered by rows, the expression level of these 12 genes could be divided into three types (Fig. 6a). The first type showed a regular pattern in which the expression levels of the DEGs were downregulated, and the expression levels were greater within LFG-0 than within SFG-0 (Fig. 6a, top). This type included three genes, *TUB6* (Gh_A07G1077), *TPS11* (Gh_D08G0936), and *ERF1* (Gh_D11G0426). The second type showed that the expression levels of DEGs were upregulated, and the expression levels within LFG-F10 were greater than those within SFG-F10 (Fig. 6a, middle). This type included 6 genes: *MES1* (Gh_D11G1910), *TUB1* (Gh_D01G0939), *TUA2* (Gh_D02G2420), *RS5* (Gh_A05G3108) and two pectin lyase (PL) family genes (Gh_D11G0237 and Gh_D12G2437). As illustrated in our expression pattern analysis, profile 0 and profile 7 were related to fiber initiation and elongation, respectively. Therefore, the highly expressed genes in the LFG might have more promoting effects. The third type was similar to the second type, but the expression levels within SFG-F10 were greater than those within LFG-F10 (Fig. 6a, bottom). *PER64* (Gh_A02G1663), Gh_A11G0965 (glycosyl hydrolase) and Gh_A05G3328 (PL) were included in this type, indicating that these three genes may negatively regulate fiber elongation.

### RNA-seq validation by qPCR

To validate the RNA-seq data, the 12 genes shown in Fig. 6a were selected for qRT-PCR analysis. A very significant correlation between the transcriptome sequencing and qPCR data was presented (Fig. 6b). Although there were certain differences in expression levels, the expression trends were essentially the same, indicating that the data obtained from the transcriptome sequencing in this study were credible.

## Discussion

### Differential energy distribution between the SFG and LFG lines

Any life activity requires energy, and the process of fiber development is no exception [22, 44]. During fiber initiation and rapid elongation, the number of transcribed genes and their expression level may also be affected by the energy distribution in fiber cells. Yoo et al. proposed a model in which carbon resources might be reallocated in cotton fibers by domestication [45]. The carbon resource reallocation hypothesis was well supported by a proteomic analysis of developing fibers [41].

In our electron and light micrographs, differences in fiber initiation between the SFG and LFG lines were demonstrated (Fig. 1). Subsequent gene expression pattern analysis indicated that the DEGs involved in fiber initiation in the SFG were more than in the LFG (Fig. 4, profile 0). Although LFG lines had a lesser fiber number than SFG lines, their FL was longer (Fig. 2), which was positively correlated with the DEGs involved in fiber elongation in the LFG (Fig. 4, profile 7). According to the hypothesis mentioned above, the SFG lines would “invest” more in fiber number, while the LFG lines would “invest” more energy in fiber length. Therefore, controlling the energy distribution in gene transcription may be critical for fiber development regulation.

### Advances in the study of fiber development mechanisms by expression profile sequencing

A transcriptome can reflect the gene expression and regulatory rules in specific tissues at the whole-genome level. With the advancement of technology, the expression profile sequencing technology has progressed from cDNA array and microarray expression analyses to transcriptome sequencing, e.g., expressed sequence tags and RNA-seq. At the beginning of 2000, using a cDNA array, Li et al. identified 14 highly expressed cDNAs in fibers from wild-type and mutant cotton plants [46]. Shi et al. constructed a cDNA library for microarray expression analysis; these authors reported that the ethylene synthesis pathway was significantly upregulated during fiber elongation and that ethylene did promote this process [27]. Later, different researchers successively studied the changes in fiber expression profiles between different cotton species [47], during various stages [48], and under domestication [31]. These findings have deepened our understanding of genes related to fiber development.

The DNA microarray method is based on the hybridization of specific probes and is subject to large background interference, which limits the number of identifiable expressed genes. As the cost of sequencing has decreased, expression profiling has entered the era of transcriptome sequencing. Compared to DNA microarrays, transcriptome sequencing has higher throughput, lower background interference, and a wider range of gene expression detection. Using DNA microarray technology, Rapp et al. compared the expression levels of 40,430 genes involved in the development of two cotton species and found that domestication altered the expression of nearly a quarter of those genes [31]. Later, the team used transcriptome sequencing to compare and analyze the transcription levels of multiple upland cotton cultivars and wild species and found that almost one-third of the genes are expressed in the fibers and that the expression of approximately 5000 genes has changed as a result of domestication [45].

With the advancement of sequencing technology and the updating of sequencing platforms, the cost of RNA-seq technology has been decreasing, and the quality of sequencing is becoming increasingly reliable. In particular, the completion of the TM-1 genome sequencing [32, 33] has provided an improved reference genome for upland cotton research, making RNA-seq the most common, conventional and effective means to study upland cotton. The present study used the most recent RNA-seq technology and obtained high-quality results with excellent clean read rates, mapping rates, GC contents, Q30 values and so forth (Additional file 3). Twenty-two DEGs involved in fiber initiation and 31 DEGs involved in fiber elongation were detected here. Some of these have been reported to be related to fiber development, whereas some are new discoveries and are discussed separately below.

### Ethylene metabolism and fiber development

Ethylene is a key regulator of cotton fiber cell growth [22, 27, 28]. The expression patterns of ethylene metabolism-related genes clearly differ between cotton species during fiber development [28, 29]. Ethylene plays a two-way regulatory role in fiber development; i.e., too much or insufficient amounts will inhibit fiber elongation, which is one of the reasons for the differences in FL [32]. In the present study, an ethylene response element binding factor (*ERF1*) was identified, and its expression level significantly differed between the two groups (Fig. 6a). The KEGG enrichment results revealed that this gene plays a role in the ethylene regulatory network of plant hormone signal transduction. The upstream factor of *ERF1* is an ethylene-insensitive protein (EIN3). Members of the EIN3 family of nuclear-localized proteins are essential the plant response to gaseous ethylene. The activation of various ethylene response genes depends on the expression of *ERF1* [49]. *ERF1* acts on downstream DNA directly, thus affecting plant growth and development. As shown in Fig. 6a, expression of the *ERF1* gene (Gh_D11G0426) was gradually downregulated in both the SFG and LFG but was highly expressed in the LFG. This finding indicated that, compared with that in the SFG, the response to ethylene in the LFG was faster and more efficient, which may be the main reason for the longer fibers in 601 LSC and J02–508.

### Tubulin and fiber development

Microtubules are an integral part of the cytoskeleton and are widespread throughout the cytoplasm. They are found in eukaryotic cells and are formed by the polymerization of globular α-tubulin and β-tubulin dimers. Microtubules, an essential structural component of fiber cells, participate in the maintenance of cell structure and form the cytoskeleton together with microfilaments [50]. When the assembly of microtubules was blocked with colchicine during fiber elongation, a large number of microtubule fragments and disordered microfibrils were visible; compared with that of the control cells, the cell wall surface of these treated cells became wrinkled, and fiber elongation was significantly inhibited [51]. In an in vitro ovule culture experiment, fiber production significantly decreased in the presence of sulfamethoxazole (a microtubule-disrupting agent) and significantly increased when treated with paclitaxel (a microtubule-stabilizing agent). The abovementioned inhibition or promotion occurred during the early developmental stage (1–3 DPA); after that stage, fiber morphology can be altered by chemical agents, but fiber numbers cannot, indicating that tubulin may play an important role in fiber initiation [52]. The tubulin content in 8–28 DPA fibers was measured using monoclonal antibodies to α- and β-tubulin, and the results showed that the content increased approximately threefold at 10–20 DPA. After 20 DPA, the proportion of tubulin content relative to the total fibrin plateaued or slightly decreased, indicating that a rapid increase in tubulin was associated with rapid fiber elongation [53]. Comparative proteomic profiling between a short-fiber mutant (*Li1*) and its wild type revealed that some cytoskeleton-related proteins in the *Li1* mutant were significantly reduced and that the actin cytoskeleton structure was severely distorted; as a result, vesicle trafficking was therefore blocked, suggesting that the short fibers of the *Li1* mutant were associated with tubulin defects [54].

In this study, three tubulin-encoding genes (*TUB6*, *TUB1*, *TUA2*) were identified (Fig. 6a). *TUB6* was highly expressed at 0 DPA, while *TUB1* and *TUA2* were highly expressed at 10 DPA, indicating that these genes play roles in fiber initiation and elongation, respectively. KEGG analysis revealed that *TUA2* and *TUB1* encode α-tubulin and β-tubulin, and their dimers can aggregate to form microtubules. Both proteins were expressed at higher levels in the LFG than in the SFG, which provided adequate microtubule protein for fiber elongation. This finding also explains why the fiber quality was better in the LFG (601 LSC and J02–508) than in the SFG lines.

### Peroxidase (POD) and fiber development

Peroxisomes, which are vesicles surrounded by a single membrane, are ubiquitous in various types of eukaryotic cells. Enzyme activity constantly changes during plant growth and development. In plant tissues, POD can convert some carbohydrates into lignin, increasing the degree of lignification; therefore, enzyme activity is generally greater in aging tissues than in young tissues. The marker enzyme of peroxisomes is catalase, whose main role is to hydrolyze hydrogen peroxide (H_2_O_2_). Numerous studies have shown that ROS play a complex regulatory role in fiber development. In in vitro ovule culture experiments, H_2_O_2_ was shown to be involved in a feedback regulatory mechanism of ethylene biosynthesis, which regulates cotton fiber development [55]. Overexpression of the *GhCaM7* gene promoted fiber elongation, and the concentration of ROS in the *GhCaM7*-overexpressing line was greater than that in the wild type, indicating that ROS are key regulators involved in fiber elongation [23]. Liu et al. detected a dramatic increase in ROS on the − 3 and − 2 DPA ovule surfaces in upland cotton fibreless mutants, indicating that homeostasis of ROS may play a key role in the regulatory mechanisms of cotton fiber development [56]. Virus-induced silencing of the *GhPK6* gene in cotton plants resulted in increased FL and a decreased accumulation of ROS, indicating that, by regulating ROS, the cotton cytoplasmic pyruvate kinase gene *GhPK6* may play an important role in cotton fiber elongation [57].

*PER64* (Gh_A02G1663) is a member of the POD family of enzymes. POD can increase the degree of lignification in fiber cells. In the present study, the *PER64* gene was highly expressed in the SFG (Fig. 6a), and its expression level gradually increased with development, revealing a significant negative regulation of fiber elongation. KEGG enrichment analysis revealed *PER64* is involved in the phenylpropanoid biosynthesis process and catalyzes the synthesis of lignin from carbohydrates. Studies have shown that the phenylpropanoid biosynthesis pathway is relatively more active in wild cotton than in domesticated cotton and is not conducive to fiber elongation, so it has been subjected to great amounts of negative selection pressure during domestication [45, 58]. Therefore, active POD in the SFG may be a reason leading to the inferiority in FL of 69–6025-12 and Liao 1779.

### Other fiber development-related genes

Three PL family genes (Gh_D11G0237, Gh_D12G2437, Gh_A05G3328) were differentially expressed during fiber elongation (Fig. 6a). Fiber primary cell walls contain high amounts of pectin, which can promote fiber elongation by regulating the production of cell wall polymers [29]. With changing pectin content, the homeostasis of cell wall polymers is altered by the differential expression of these three genes. These facts can somewhat explain the differences in FL between the SFG and LFG lines. Some carbohydrate metabolism-related genes were also identified (Fig. 6a). For example, the protein encoded by *TPS11* (Gh_D08G0936) catalyzes the synthesis of trehalose from glucose, and that encoded by *RS5* (Gh_A05G3108) catalyzes the production of raffinose from galactosides and sucrose. Sugars not only provide energy for cell development but also serve as substrates for the biosynthesis of cell wall polymers such as cellulose and pectin; therefore, sugars are closely related to fiber development [21, 59].

In total, 53 DEGs were identified through the KEGG enrichment analysis (Table 3). The discussion of the genes with known functions above indicates that our RNA-seq results are reliable. The remaining genes in Table 3 would be good candidates for revealing the mechanisms of fiber initiation and elongation.

## Conclusions

In this study, differences in the fiber initiation and elongation stages between long- and short-fiber cotton lines were determined based on phenotypic observations. The developmental dynamics of the cotton fiber transcriptomes of these lines was the comparatively analyzed with RNA-seq. Gene expression pattern analysis of the DEGs between developmental stages revealed that profile 0 and profile 7 were enriched both in the SFG and LFG. Twenty-two genes were identified from profile 0 (related to fiber initiation), in which *ERF1* was involved in ethylene metabolism and was expressed at a higher level in the LFG than in the SFG, which affected fiber initiation. Thirty-one genes were identified from profile 7 (related to fiber elongation), in which *TUA2* and *TUB1* were involved in microtubule synthesis and were expressed at a higher level in the LFG than in the SFG, while *PER64*, which encoded a POD, was expressed at a higher level in the SFG. From a functional standpoint, microtubules were the exact structural component necessary for fiber elongation, and by ultimately accelerating cell lignification, POD was not conducive to fiber elongation. Therefore, the differential expression of these genes may be the main reason leading to phenotypic differences in the fibers.

The results of this study have increased the understanding of relevant metabolic pathways involved in the process of fiber initiation and elongation. It is the first step in screening candidate genes for genetic improvement of cotton fiber. This work supplements current knowledge in this area and can guide future research.

## Additional files

Additional file 1: Detailed bioinformatics analytical process. (DOCX 30 kb)

Additional file 2: Primers used for qRT-PCR. (XLSX 11 kb)

Additional file 3: Data output quality list. (XLSX 18 kb)

Additional file 4: Violin diagram of FPKM values. The abscissa shows the sample name, and the ordinate represents the log_10_(FPKM + 1). The dotted line in the figure indicates that FPKM = 1. The violin plots for each sample correspond to five statistical results (from top to bottom: maximum, upper quartile, median, lower quartile and minimum). The width of each violin represents the number of genes at that expression level. The FPKM values are the average of two biological replicates. (JPG 38 kb)

Additional file 5: Pearson correlation coefficient analysis. R^2^: the square of the Pearson correlation coefficient. (JPG 166 kb)

Additional file 6: Principal component analysis. The meaning of each letter and number in the sample name is the same as that in Table 2 and Additional file 3. (JPG 115 kb)

Additional file 7: Identification of DEGs from different comparisons. (XLSX 7975 kb)

Additional file 8: Cluster analysis of all DEGs from 8 samples. In the overall FPKM hierarchical clustering map, red indicates highly expressed genes, and blue indicates genes expressed at low levels. The meaning of each character and number in the sample name is the same as that in Additional file 4. (TIF 64 kb)

Additional file 9: GO categories for DEGs within significantly enriched profiles. (XLSX 959 kb)

Additional file 10: KEGG enrichment results. (XLSX 20 kb)

## Data Availability

Sequence data of 16 RNA-Seq have been uploaded to NCBI Sequence Read Archive (SRA) database (https://www.ncbi.nlm.nih.gov/sra/), and the SRA number was SRP116606.

## Abbreviations

DEGs: Differentially expressed genes
DPA: Day post anthesis
EIN3: Ethylene-insensitive protein
ERF1: Ethylene responsive element binding factor 1
FL: Fiber length
FPKM: Fragments Per Kilobase of transcript sequence per Millions base pairs sequenced
GA: Gibberellins
GO: Gene Ontology
KEGG: Kyoto Encyclopedia of Genes and Genomes
LFG: Long fiber group
LSC: Long-Staple cotton
MES1: Methyl esterase 1
PCA: Principal component analysis
PER64: Pectin lyase-like superfamily protein
PL: Pectin lyase
POD: Peroxidase; ROS Reactive oxygen species
RS5: Raffinose synthase family protein
SFG: Short fiber group
STEM: Short time-series expression miner
TPS11: Trehalose phosphatase/synthase 11
TUA2: Tubulin alpha-2 chain
TUB1: Tubulin beta-1 chain
TUB6: beta-6 tubulin
