# Supplementary material for: Transcriptome analysis reveals differences in the mechanisms of fiber initiation and elongation between long- and short-fiber cotton (Gossypium hirsutum L.) lines
Source: BMC Genomics. 2019 Aug 5;20:633. doi: 10.1186/s12864-019-5986-5 (PMC6683361; doi:10.1186/s12864-019-5986-5)
Supplement: Supplementary file 1 — Detailed bioinformatics analytical process. (DOCX 30 kb) [file 12864_2019_5986_MOESM1_ESM.docx]

**Additional file 1: Detailed bioinformatics analysis process**

**Data quality control**

Raw data (raw reads) in FASTQ format were first processed through in-house Perl scripts. In this step, clean reads were obtained by removing reads containing adapter, reads containing ploy-N and low-quality reads from raw data. At the same time, the Q20, Q30 and GC contents of the clean data were calculated. All the downstream analyses were based on clean data of high quality.

**Reads mapping to the reference genome**

The *Gossypium hirsutum* TM-1 reference genome [1] and gene model annotation files were downloaded from the CottonGen database (http://www.cottongen.org) directly. The index of the reference genome was built using Bowtie v2.2.3 [2], and paired-end clean reads were aligned to the reference genome using TopHat v2.0.12. We selected TopHat as the mapping tool because TopHat can generate a database of splice junctions based on the gene model annotation file and thus yield a better mapping result than other nonsplice mapping tools [3, 4].

**Quantification of gene expression level**

HTSeq v0.6.1 was used to count the read numbers mapped to each gene [5]. Then the FPKM of each gene was calculated based on the length of the gene and read count mapped to this gene. FPKM considers the effects of sequencing depth and gene length on the read count at the same time and is currently the most commonly used method for estimating gene expression levels [6].

**Differential expression analysis**

Differential expression analysis was performed using the DESeq R package (1.18.0). DESeq provides statistical routines for detecting differential expression in digital gene expression data using a model based on the negative binomial distribution. The resulting P-values were adjusted using the Benjamini and Hochberg approach for controlling the false-discovery rate (FDR). Genes with an adjusted P-value < 0.05 found by DESeq and |log_2_(fold change)| > 1 were assigned as differentially expressed.

**Function method description**

GO enrichment analysis provides all GO terms that are significantly enriched among DEGs compared to the genome background, and it filters the DEGs according to their biological functions. First, all DEGs were mapped to GO terms in the GO database (<http://www.geneontology.org/>). Gene numbers were calculated for every term. Significantly enriched GO terms among DEGs compared to the genome background were defined by the hypergeometric test. The calculated P-value was put through FDR correction, taking FDR ≤ 0.05 as a threshold. GO terms meeting this condition were defined as significantly enriched GO terms of DEGs. This analysis was able to recognize the main biological functions of the DEGs.

KEGG is the major public pathway-related database. Pathway enrichment analysis identified significantly enriched metabolic pathways or signal transduction pathways in DEGs compared with the whole-genome background. Significantly enriched pathways of DEGs compared to the genome background were defined by the hypergeometric test. The calculated P-value was put through FDR correction, taking FDR ≤ 0.05 as a threshold. Pathways meeting this condition were defined as significantly enriched pathways among the DEGs.

Gene expression pattern analysis was performed by STEM software [7]. The parameters were set as follows: Maximum Unit Change in model profiles between time points was 1; Maximum output profile number was 20 (similar profiles will be merged); Minimum ratio of fold change of DEGs was no less than 2.0.

All the analyses above were performed using OmicShare tools, a free online platform for data analysis (www.omicshare.com/tools).

**References**

1. Zhang TZ, Hu Y, Jiang WK, Fang L, Guan XY, Chen JD, Zhang JB, Saski CA, Scheffler BE, Stelly DM *et al*: **Sequencing of allotetraploid cotton (*Gossypium hirsutum L*. acc. TM-1) provides a resource for fiber improvement**. *Nature biotechnology* 2015, 33(5):531-537.

2. Langmead B, Salzberg SL: **Fast gapped-read alignment with Bowtie 2**. *Nature methods* 2012, 9(4):357-359.

3. Trapnell C, Pachter L, Salzberg SL: **TopHat: discovering splice junctions with RNA-Seq**. *Bioinformatics* 2009, 25(9):1105-1111.

4. Trapnell C, Roberts A, Goff L, Pertea G, Kim D, Kelley DR, Pimentel H, Salzberg SL, Rinn JL, Pachter L: **Differential gene and transcript expression analysis of RNA-seq experiments with TopHat and Cufflinks**. *Nature Protocol* 2012, 7(3):562-578.

5. Anders S, Pyl PT, Huber W: **HTSeq--a Python framework to work with high-throughput sequencing data**. *Bioinformatics* 2015, 31(2):166-169.

6. Trapnell C, Williams BA, Pertea G, Mortazavi A, Kwan G, van Baren MJ, Salzberg SL, Wold BJ, Pachter L: **Transcript assembly and quantification by RNA-Seq reveals unannotated transcripts and isoform switching during cell differentiation**. *Nature biotechnology* 2010, 28(5):511-515.

7. Ernst J, Bar-Joseph Z: **STEM: a tool for the analysis of short time series gene expression data**. *BMC bioinformatics* 2006, 7:191.
