# Supplementary figures and images for: Transcriptome analysis reveals differences in the mechanisms of fiber initiation and elongation between long- and short-fiber cotton (Gossypium hirsutum L.) lines
Source: BMC Genomics. 2019 Aug 5;20:633. doi: 10.1186/s12864-019-5986-5 (PMC6683361; doi:10.1186/s12864-019-5986-5)

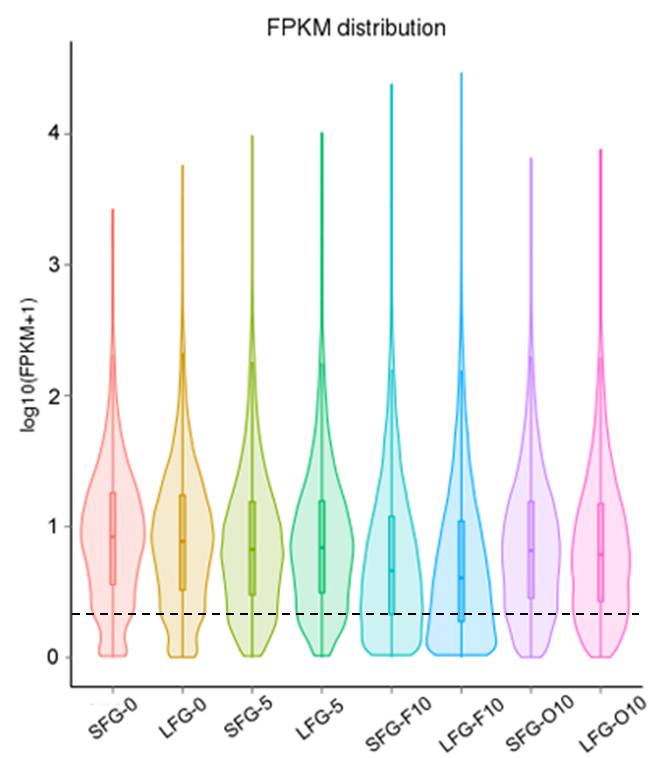

Supplement: Supplementary file 4 — Violin diagram of FPKM values. The abscissa shows the sample name, and the ordinate represents the log10(FPKM + 1). The dotted line in the figure indicates that FPKM = 1. The violin plots for each sample correspond to five statistical results (from top to bottom: maximum, upper quartile, median, lower quartile and minimum). The width of each violin represents the number of genes at that expression level. The FPKM values are the average of two biological replicates. (JPG 38 kb) [file 12864_2019_5986_MOESM4_ESM.jpg]

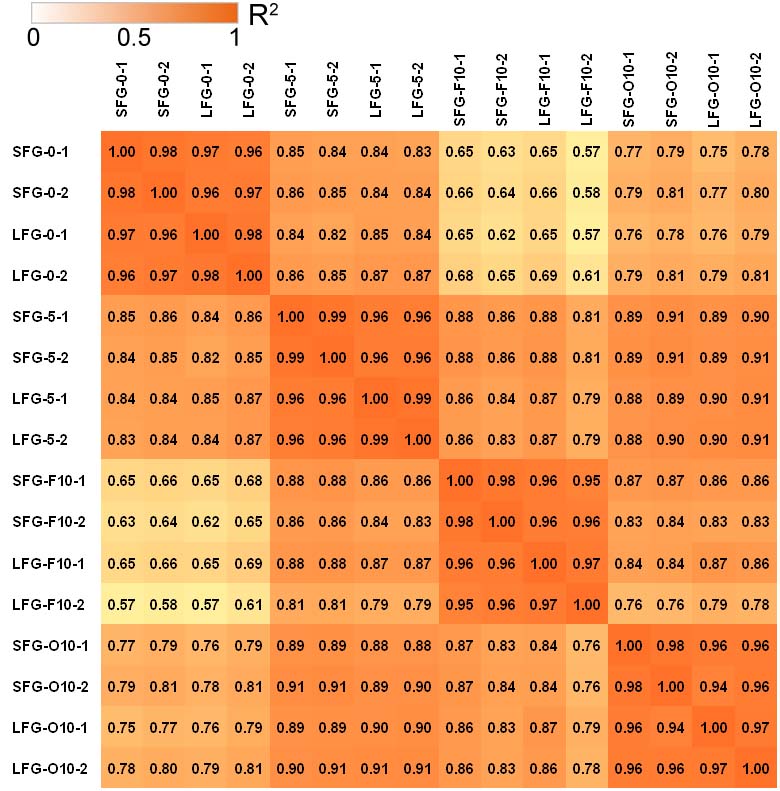

Supplement: Supplementary file 5 — Pearson correlation coefficient analysis. R2: the square of the Pearson correlation coefficient. (JPG 166 kb) [file 12864_2019_5986_MOESM5_ESM.jpg]

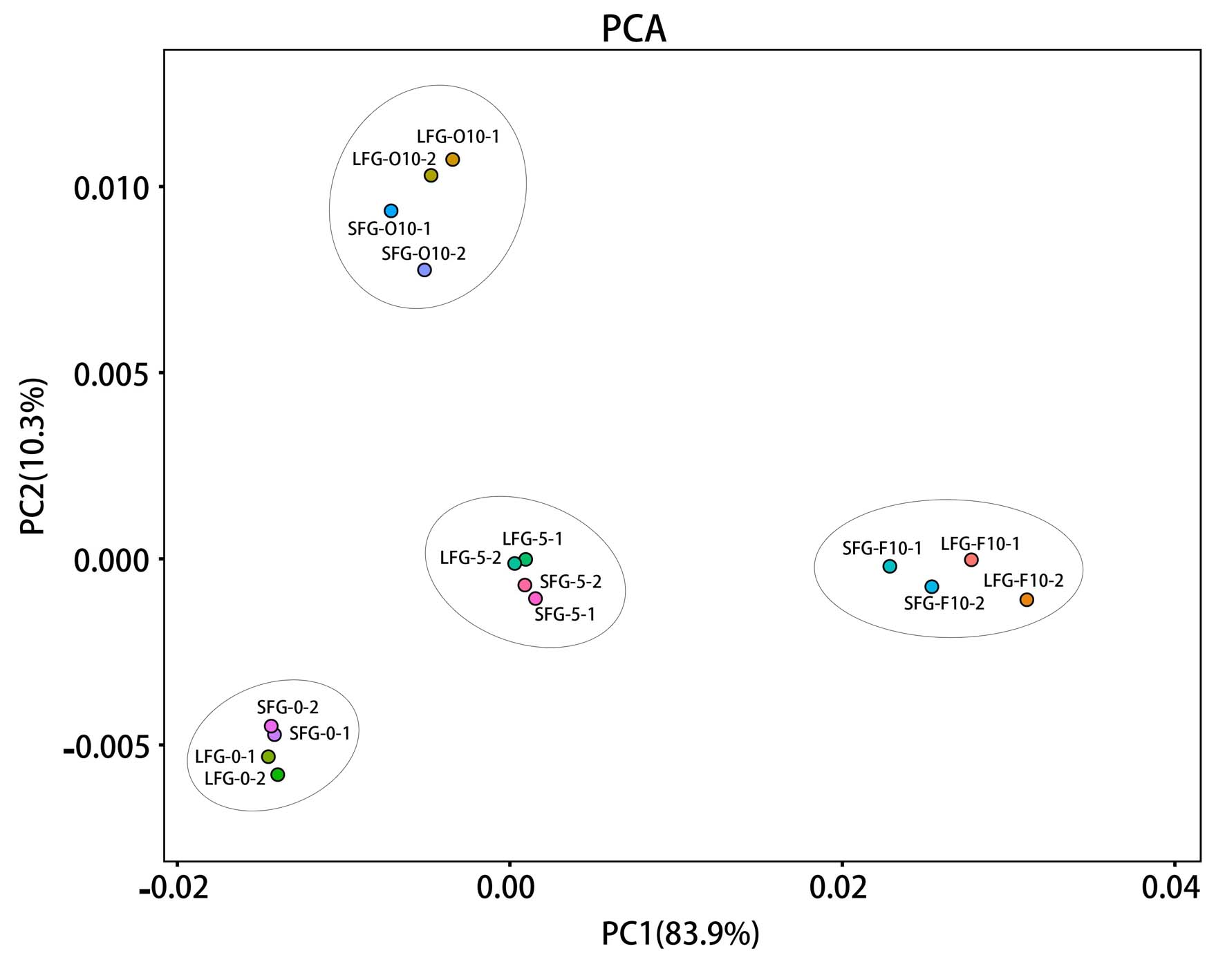

Supplement: Supplementary file 6 — Principal component analysis. The meaning of each letter and number in the sample name is the same as that in Table 2 and Additional file 3. (JPG 115 kb) [file 12864_2019_5986_MOESM6_ESM.jpg]

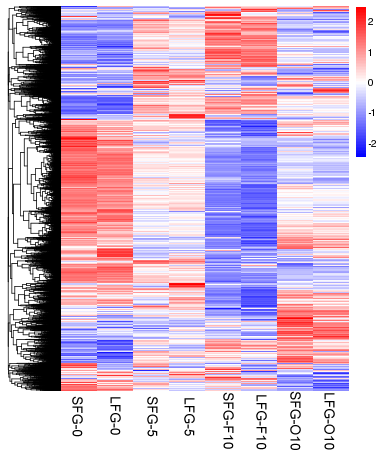

Supplement: Supplementary file 8 — Cluster analysis of all DEGs from 8 samples. In the overall FPKM hierarchical clustering map, red indicates highly expressed genes, and blue indicates genes expressed at low levels. The meaning of each character and number in the sample name is the same as that in Additional file 4. (TIF 64 kb) [file 12864_2019_5986_MOESM8_ESM.tif]
